# Trypanosomal histone γH2A and the DNA damage response

**DOI:** 10.1016/j.molbiopara.2012.01.008

**Published:** 2012-05

**Authors:** Lucy Glover, David Horn

**Affiliations:** London School of Hygiene & Tropical Medicine, Keppel Street, London WC1E 7HT, UK

**Keywords:** Checkpoint, Chromatin, γH2AX, I-*Sce*I, MMS, Repair

## Abstract

DNA damage and repair in trypanosomatids impacts virulence, drug resistance and antigenic variation but, currently, little is known about DNA damage responses or cell cycle checkpoints in these divergent protozoa. One of the earliest markers of DNA damage in eukaryotes is γH2A(X), a serine phosphorylated histone H2A (variant). Here, we report the identification and initial characterization of γH2A in *Trypanosoma brucei*. We identified Thr^130^ within the replication-dependent histone H2A as a candidate phosphorylation site and found that the abundance of this trypanosomal γH2A increased *in vivo* in response to DNA damage. Nuclear γH2A foci mark the sites of putative natural replication fork stalling, sites of meganuclease-induced DNA double strand breaks and sites of methyl methanesulphonate-induced DNA damage. Naturally occurring and meganuclease-induced γH2A and RAD51 double-positive repair foci are typically found in S-phase or G_2_ nuclei. The results link trypanosomal γH2A, with an unusual histone modification motif, to DNA damage sensing and mitotic checkpoint signaling.

## Graphical abstract

**Graphical abstract:**

**Figure:**
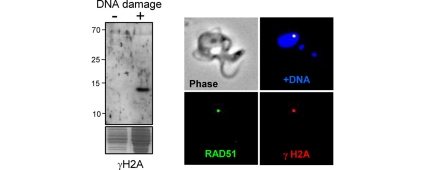

**Highlights:**

► Histone H2A phosphorylated at Thr130 is identified as trypanosomal γH2A. ► γH2A nuclear foci mark the putative sites of replication fork stalling. ► γH2A nuclear foci form in response to meganuclease or chemical-induced DNA damage. ► Trypanosomal γH2A and RAD51 positive damage and repair foci are most often seen in S-phase and G_2_. ► The findings implicate γH2A in mitosis-entry checkpoint signaling.

## 1 Introduction

DNA rearrangement in trypanosomatids can bring about changes in virulence and drug resistance and is well known for its role in switching variant surface glycoprotein expression and antigenic variation in *Trypanosoma brucei* [1]. Efficient homologous recombination in trypanosomatids is also exploited for targeting recombinant gene expression cassettes to specific chromosomal loci. Thus, it will be important to improve our understanding of the mechanisms regulating trypanosomal DNA repair.

Homologous recombination dominates DNA double strand break repair in *T. brucei* [2] and microhomology-mediated end joining also operates [3], while non-homologous end-joining has not been reported. A number of *T. brucei* factors have demonstrated roles in homologous recombination [1], and site-specific cleavage, following inducible expression of the yeast I-*Sce*I meganuclease, has proven to be a powerful tool, allowing repair monitoring [2], investigation of mechanisms of antigenic variation [4], and genetic dissection of repair pathways [3]. Studies in this area are restricted, however, due to the relative paucity of reagents available to investigate DNA damage and repair processes in trypanosomatids.

Eukaryotic nuclear DNA is packaged with nucleosomes to form chromatin. The octameric nucleosomes are comprised of two units of each core histone, H2A, H2B, H3 and H4, and approximately 150 bp of DNA is wrapped on the outer face of each of these nucleosomes. Histone post-translational modification or replacement by histone variants can lead to the specialization of chromatin domains to facilitate processes such as DNA repair, chromosome segregation or transcription control. Thus, chromatin components and chromatin structure have a major impact on the DNA damage response through cell cycle checkpoint control and the recruitment of repair factors [5]. A number of histone modifications and variants have been characterized in *T. brucei* [6]. However, one of the most prominent and earliest markers of DNA damage in eukaryotes, γH2A(X), has not been described in any trypanosomatid.

γH2AX in mammalian cells is a Ser^139^ phosphorylated version of the histone variant H2AX [7]. In *Drosophila melanogaster*, the equivalent of γH2AX is a Ser^137^ phosphorylated version of H2AZ [8], a histone variant typically involved in transcription control. In the budding yeast, *Saccharomyces cerevisiae* [9,10], and in the fission yeast, *Schizosaccharomyces pombe* [11] γH2A is a Ser^128/129^ phosphorylated version of the major, replication-dependent histone. Cells with H2AX mutations [12,13] or lacking the relevant Ser residue in H2A [10,11] display a range of DNA repair defects. At least three phosphoinositide-3-kinase-related protein kinases (PIKKs) can phosphorylate and modify the function of H2A(X) but these kinases also modify other repair effectors, making it difficult to dissect the role of each factor [5]. DNA damage triggers the local accumulation or generation of γH2A(X) which, in turn, contributes to a DNA damage signaling cascade and cell-cycle arrest. To achieve this, the phosphorylated histone tail of γH2A(X) recruits additional repair-related factors [14,15], including mediator of DNA damage checkpoint protein 1, MDC1 [16], a histone H4 acetyltransferase complex [17,18] and cohesin [19], resulting in the generation of an extended domain [20] of less condensed, more accessible chromatin. We now report the identification and initial characterization of *T. brucei* γH2A.

## 2 Materials and methods

### 2.1 Strains

*T. brucei* Lister 427, MITat1.2 (clone 221a), bloodstream form cells were grown in HMI-11 and transformed as described [21]. MMS (Sigma) was applied to *T. brucei* cultures at 0.0003% for 24 h. Phleomycin (Sigma) was applied at 1 μg ml^−1^ for 18 h. For meganuclease-induction, tetracycline (Sigma) was applied at 1 μg ml^−1^ for 12 h. RAD51, with Green Fluorescent Protein fused to the *C*-terminus, was expressed using the pNAT^RAD51-GFP^ construct, a derivative of pNAT^xGFP^ [22]. pNAT^RAD51-GFP^ was linearized by digestion with *Nsi*I prior to transfection. Oligonucleotide details are available on request.

### 2.2 Antisera and western blotting

Anti-γH2A antisera were raised in rabbits according to a 90-day protocol using a KLH-conjugated phospho-peptide, C-KHAKA[pT]PSV (Open BioSystems). Antiserum with an enzyme-linked immunosorbent assay (ELISA) titre of 1:204,800 was affinity purified using the corresponding peptides (Open Biosystems). Western blotting was carried out as described [2], except that 1% (v/v) phosphatase inhibitors (Sigma) were added to the lysis buffer and samples were separated on a 15% SDS-PAGE gel. Primary *T. brucei* γH2A antibody was used at a 1:200 dilution and secondary goat anti-rabbit IgG HRP (Bio-Rad) was used at a 1:2000 dilution. For the peptide competition assay, primary antibody was pre-incubated with 40 ng ml^−1^ of the appropriate peptide in wash buffer for 1 h at room temperature prior to incubation with the immunoblot.

### 2.3 Cell cycle analysis and immunofluorescence microscopy

Immunofluorescence detection and imaging were carried out as described [2]. Primary γH2A antibody and secondary fluorescein-conjugated goat anti-rabbit (Pierce) were used at a 1:100 dilution. DNA was stained with 4′,6-diamino-2-phenylindole (DAPI, Vector Laboratories) prior to fluorescence microscopy. All counts for the quantitative analysis of cell cycle phases or proportions of cells with RAD51 and/or γH2A foci were carried out by both of us. Images were captured using a Nikon Eclipse E600 epifluorescence microscope in conjunction with a Coolsnap FX (Photometrics) charge-coupled device (CCD) camera and processed in Metamorph 5.0 (Photometrics).

## 3 Results and discussion

### 3.1 A putative γH2A-like phosphorylation site at the C-terminus of trypanosomal histone H2A

**Fig. 1:**
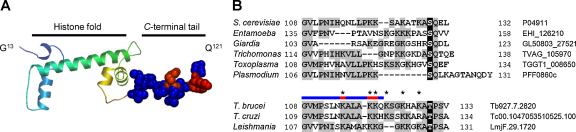
Thr^130^ is a putative γH2A-type kinase substrate in trypanosomal histone H2A. (A) A homology model of *T. brucei* histone H2A (residues 13–121) was generated using SWISS-MODEL [31] and shows how the *C*-terminal tail extends beyond the core histone-fold domain. The space-filling portion of the model represents residues 108–121 (see colored bar in B). (B) Alignment of the *C*-termini of major histone H2A sequences from budding yeast and parasitic protozoa. The S of the SQ motif and the putative equivalent T are on a black background while other residues that are identical in more than two sequences are shaded. It is notable that this region of H2A in *T. brucei* appears to be hyper-acetylated [32], as indicated by asterisks. Full sequence information can be retrieved from the NCBI (ncbi.nlm.nih.gov/) or eupathdb (eupathdb.org/) databases using the accession numbers on the right. Note that the numbering for all sequences, except for *S. cerevisiae*, excludes the initiator methionine. (For interpretation of the references to color in this figure legend, the reader is referred to the web version of the article.)

Trypanosomatid genomes encode a replication-dependent [23] histone H2A and an H2AZ variant [24,25]. H2AZ is encoded by a single-copy gene on chromosome 7, while thirteen tandem copies of the histone *H2A* gene are annotated; these are also on chromosome 7 but at a distal locus relative to *H2AZ*. All predicted *T. brucei* H2A protein sequences are identical in the genome reference strain [26] and in the current Lister 427 experimental strain. We examined the H2A and H2AZ sequences for a candidate γH2A-like potential phosphorylation site, characterized in other eukaryotes by a conserved ‘SQ-motif’ and found within two residues of the *C*-terminus. Trypanosomatid histones are probably the most divergent eukaryotic histones known [24,27] and, consistent with this divergence, we failed to identify an SQ-motif close to the *C*-terminus of trypanosomal H2A (Fig. 1) or H2AZ (not shown). We did, however, identify a candidate equivalent phosphorylation substrate, a threonine residue within three residues of the *C*-terminus of all trypanosomal H2A sequences. Fig. 1A shows the position of the *T. brucei* histone H2A *C*-terminal tail in relation to the conserved histone-fold domain; these histone tails extend beyond the core of the nucleosome. Fig. 1B shows the aligned histone H2A sequences, highlighting the location of the Ser of the SQ-motif identified in all other protozoal H2A sequences examined and the conserved Thr residue in trypanosomal H2A, Thr^130^ in *T. brucei*.

### 3.2 H2A-Thr^130^ is phosphorylated *in vivo* in response to DNA damage

**Fig. 2:**
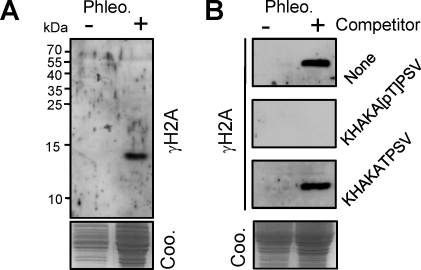
Trypanosomal γH2A is histone H2A phosphorylated at Thr^130^. (A) A western blot of whole *T. brucei* lysate before and after phleomycin (Phleo) exposure shows that γH2A levels increase following DNA damage. The coomassie (Coo.) panel shows loading. (B) The peptide competition assay demonstrates the specificity of the antibody. Other details are as for A.

To ask whether H2A-Thr^130^ is phosphorylated *in vivo*, antibodies were raised to the cognate phospho-peptide. A lysate from cells exposed to the DNA damaging agent, phleomycin, was compared to a lysate from untreated cells using western blot analysis. This revealed a signal consistent with H2A-Thr^130^ phosphorylation that was specifically induced by phleomycin; the predicted mass of *T. brucei* H2A is 14.2 kDa (Fig. 2A). A peptide competition assay was then used to demonstrate the specificity of the antibody; only the phosphorylated peptide was able to deplete the damage-inducible γH2A-like signal (Fig. 2B). We conclude that *T. brucei* γH2A, and very likely γH2A in other trypanosomatids, is the major, replication-dependent H2A phosphorylated on the most *C*-terminal threonine.

### 3.3 Focal accumulation of trypanosomal γH2A increases in response to chemical or enzymatic DNA damage

**Fig. 3:**
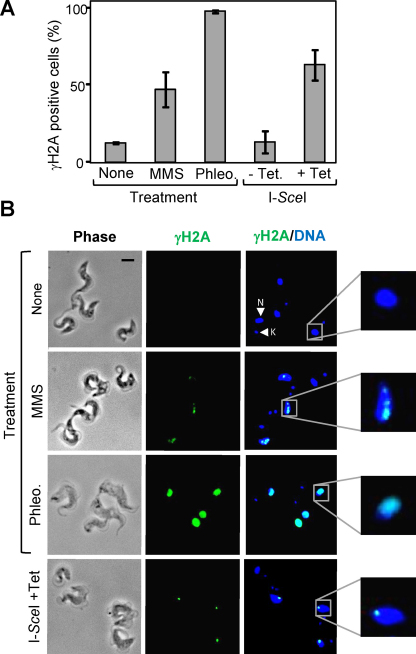
*T. brucei* γH2A nuclear signals increase in response to DNA damage. (A) Chemical and enzyme-induced damage leads to an increase in the proportion of γH2A positive cells, as determined by immunofluorescence microscopy. I-*Sce*I; cells engineered with an I-*Sce*I cleavage site on chr. 11 and a Tet-inducible I-*Sce*I gene. *n* = 200 for each sample. Error bars represent one standard deviation. (B) Representative immunofluorescence microscopy images reveal γH2A positive nuclei. N, nucleus; K, kinetoplast; Scale bar = 5 μm.

We next used light microscopy to visualize and quantify the consequences of exposure to two distinct DNA damaging agents or to meganuclease-induced breaks. Anti γH2A fluorescence microscopy revealed signals in approximately 10% of unperturbed wild-type cells and a substantially increased proportion of cells with these signals following exposure to DNA damaging agents (Fig. 3A). Methyl methanesulphonate (MMS) exposure typically produced multiple foci per nucleus in close to 50% of cells, while phleomycin exposure typically produced whole nuclear staining in close to 100% of cells (Fig. 3B).

Inducible expression of the I-*Sce*I meganuclease in *T. brucei* with a single engineered I-*Sce*I cleavage site allows the analysis of responses to single, site-specific DNA double strand breaks [2,4]. We examined γH2A signals with and without induction of a meganuclease-mediated break in the core of chromosome 11 [2]. These breaks elicited a substantial increase in the proportion of cells displaying γH2A signals (Fig. 3A). In this case, more than 50% of nuclei were typically characterized by a single γH2A focus (Fig. 3A and B). Thus, γH2A signals were specific to the nuclear compartment, as expected, and were typically characterized by sub-nuclear foci.

### 3.4 γH2A foci are typically observed in cells in S-phase or G_2_

**Fig. 4:**
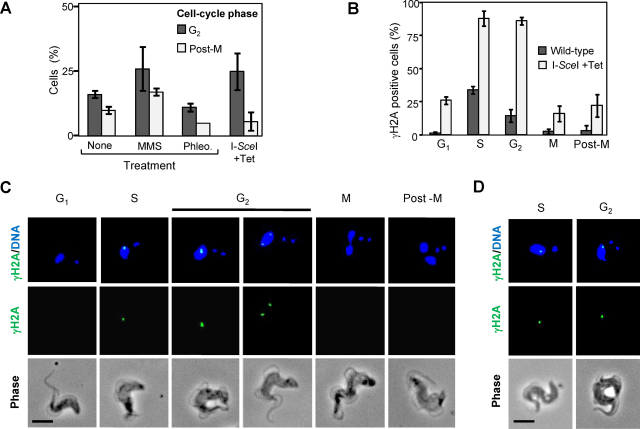
*T. brucei* γH2A DNA repair foci are enriched in S-phase and G_2_. (A) Cell-cycle distribution is perturbed by MMS and by DNA double strand breaks. In DAPI-stained cells, a single nucleus and two kinetoplasts correspond to G_2_ while two nuclei and two kinetoplasts indicate post-mitosis (post-M) [28]. I-*Sce*I; cells engineered with an I-*Sce*I cleavage site on chr. 11 and a Tet-inducible I-*Sce*I gene. *n* = 200 for each sample. Error bars represent one standard deviation. (B) Naturally occurring and meganuclease-induced γH2A foci are enriched in S-phase and G_2_. For this analysis G_2_ and post-M cells are defined as described above. In addition, we document cells with a single nucleus and a single rounded kinetoplast, which correspond to G_1_; cells with a single nucleus and an elongated kinetoplast, which correspond to S-phase [28]; and cells with bi-lobed nuclear DNA staining and two kinetoplasts, which correspond to mitosis. *n* ≥ 50 for each bar. Error bars represent one standard deviation. (C) Gallery of representative immunofluorescent microscopy images showing meganuclease induced focal accumulation of γH2A during the cell cycle. Scale bar = 5 μm. (D) Representative immunofluorescent microscopy images showing naturally occurring focal accumulation of γH2A. Scale bar = 5 μm.

Delayed progression to mitosis and focal accumulation of the RAD51 recombinase was previously reported in response to a meganuclease-mediated break in the core of chromosome 11 [2], but little else is known about DNA damage responsive checkpoints in trypanosomatids. We, therefore, examined and quantified γH2A signals at different phases of the cell cycle. Nuclear and mitochondrial (kinetoplast) DNA, stained with DAPI, provide excellent cytological markers that define position in the nuclear cycle in cells from asynchronous *T. brucei* cultures [28]. Initially, we used DAPI-staining to determine whether DNA damage perturbs cell cycle progression (Fig. 4A). MMS-treated cells displayed delayed progression to mitosis and delayed progression to cytokinesis, phleomycin-treated cells displayed no major cell-cycle disturbances and cells with a meganuclease-induced break displayed the expected delayed progression to mitosis [2]. Although MMS is thought to cause replication fork stalling [29] and phleomycin is thought to cause DNA double strand breaks [30], mechanisms of chemical-induced DNA damage are not fully understood. It is also a challenge to quantify the number of chemical-induced breaks per cell, and a high proportion of chemical-damaged cells may harbor unrepairable lesions. Thus, whole nuclear γH2A staining in the absence of major cell-cycle disturbance and an apparent delayed progression to cytokinesis were specific to phleomycin and MMS-treated cells, respectively. We, therefore, focused on natural lesions and defined meganuclease-induced breaks for further cell-cycle analysis. Single meganuclease-induced breaks at the chromosome 11 locus studied here are successfully repaired by allelic homologous recombination in more than 50% of cells [2].

Although the proportions of nuclei with γH2A foci were substantially different in unperturbed wild-type compared to meganuclease-damaged cells, in both cases, these foci were most commonly found in nuclei in S-phase and G_2_ (Fig. 4B). Cells proceeding to mitosis or through cytokinesis or G_1_ most often lack γH2A foci. In meganuclease-damaged cells, consistent with highly efficient induction of DNA double strand breaks, approximately 90% of S-phase and G_2_ nuclei contain at least one γH2A focus. The panel of images in Fig. 4C shows a typical pattern of γH2A focus formation and dissolution during the cell cycle following meganuclease-induced lesion formation. Since each cell contains two meganuclease cleavage sites post DNA replication, we examined the proportions of these nuclei with a pair of distinct foci (data not shown). As expected, the proportion of cells with two distinct foci was highest, reaching approximately 25%, at the S and G_2_-phases of the cell cycle. However, we were unable to unambiguously assign any pair of foci to meganuclease-induced lesions due to the background of naturally occurring lesions. Fig. 4D shows the appearance of γH2A foci due to these naturally occurring lesions; these foci were indistinguishable from meganuclease-induced foci in S-phase but typically appeared to be smaller than meganuclease-induced foci in G_2_-phase. This latter observation may reflect more rapid repair of natural lesions, allowing more rapid disassembly of repair foci.

### 3.5 γH2A and RAD51 colocalize at DNA repair foci

**Fig. 5:**
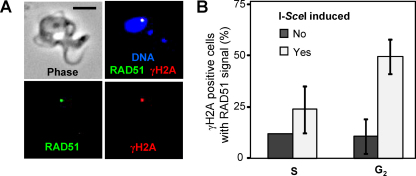
*T. brucei* γH2A and RAD51^GFP^ foci colocalize. (A) Images of a representative cell show γH2A and RAD51^GFP^ colocalization. Cells engineered with an I-*Sce*I cleavage site on chr. 11 and a Tet-inducible I-*Sce*I gene were exposed to Tet prior to analysis. γH2A was detected by immunofluorescence while GFP fluorescence was detected directly. Scale bar = 5 μm. (B) Quantitative analysis of γH2A and RAD51^GFP^ colocalization in cells with or without meganuclease induced DNA double-strand breaks. S and G_2_ phases of the cell cycle are as described in the legend to Fig. 4A and B. *n* = 100 for each bar. Error bars represent one standard deviation.

We next asked whether γH2A foci represent the sites of focal accumulation of the RAD51 recombinase. Cells with an I-*Sce*I cleavage site on chr. 11 and a Tet-inducible I-*Sce*I gene were engineered to express a copy of RAD51 with Green Fluorescent Protein (GFP) fused to the *C*-terminus. Cells with and without meganuclease-induced breaks were then examined for γH2A and RAD51 signals by microscopy. Fig. 5A shows a representative example of a cell with a meganuclease-induced DNA double strand break and overlapping γH2A and RAD51 signals. Indeed, cells with positive signals for both proteins consistently exhibited colocalized foci; we captured a set of ten overlayed images and observed colocalization in every case (data not shown). Thus, sites of focal accumulation of γH2A do correspond to sites of focal accumulation of RAD51^GFP^. It was also noted that these signals were almost always at the edge of the DAPI-stained nuclear region (also see Figs. 3B, 4C and D), suggesting migration of damaged DNA to the nuclear periphery. To further quantify γH2A and RAD51 signals, we counted γH2A positive nuclei that were also RAD51 positive; RAD51-positive nuclei were always γH2A-positive. Approximately 10% of γH2A-positive nuclei were RAD51 positive in S-phase and G_2_ cells prior to meganuclease induction (Fig. 5B). In contrast, following meganuclease-induction, approximately 25% and 50% of γH2A-positive nuclei were RAD51 positive in S-phase and G_2_ cells, respectively (Fig. 5B). As suggested above, this may reflect more rapid repair and disassembly of foci at naturally occurring lesions. Thus, the time taken to repair double-strand breaks may allow for the accumulation of an increased dosage of both γH2A and RAD51. Taken together, the results also support the idea that γH2A and RAD51 foci mark naturally occurring and meganuclease-induced DNA damage and repair sites.

## 4 Conclusions

Our findings indicate that γH2A foci are formed in response to natural DNA lesions, meganuclease-mediated DNA double strand breaks, and chemical DNA damage. Naturally occurring and meganuclease-induced foci are strongly enriched in S-phase and G_2_. Most natural DNA damage probably occurs during DNA replication, reflecting stalled, or possibly collapsed, replication forks [5], and it is notable that these lesions are not typically identified by RAD51 immunofluorescence [2]. Thus, a sharp increase in naturally occurring γH2A foci at S-phase and reduction in the number of cells with these foci in G_2_ (Fig. 4B), along with few visible RAD51 foci, suggest that natural lesions are rare outside of S-phase and rapidly repaired in S-phase and/or G_2_. In contrast, it seems likely that artificial meganuclease expression introduces breaks at all stages of the cell-cycle. Consistent with this, meganuclease-induced γH2A foci are seen in all cell-cycle phases examined (Fig. 4B), but a major increase in focus formation during S-phase and G_2_ suggests specific activation of DNA damage surveillance and repair activities in these phases. The persistence through G_2_ and increased intensity of meganuclease-induced γH2A and RAD51 foci suggests that more time is required to repair these DNA double strand breaks. In any case, successful repair leads to focus disassembly, mitosis-entry checkpoint release and progression to mitosis. A pre S-phase checkpoint may also operate but we are currently unable to distinguish between early and late G_1_ cells to test this possibility.

γH2A(X) in eukaryotes, ranging from humans to yeast, is a serine-phosphorylated histone H2A variant or the major, replication-dependent histone H2A. The latter is the case in budding yeast and also appears to be the case in several protozoal species. In *T. brucei*, and probably in other trypanosomatids, γH2A is, unusually, threonine-phosphorylated H2A. The increase in trypanosomal γH2A repair foci in response to DNA damage indicates that at least one trypanosomal kinase senses DNA double strand breaks and responds by phosphorylating histone H2A. The highly specific cell-cycle distribution of the resulting specialized chromatin foci reflects generation of a checkpoint signal that blocks progression to mitosis. Identification of trypanosomal γH2A as a prominent repair marker will facilitate studies on DNA recombination and repair and help to develop our understanding of the mechanisms by which these processes enhance parasite virulence.
